# Accuracy assessment of the Global Forest Change tree cover loss by visual interpretation and comparison with forestry logging records in Slovakia

**DOI:** 10.1007/s10661-026-15131-5

**Published:** 2026-03-12

**Authors:** Michal Druga, Vladimír Šagát, Adam Rusinko

**Affiliations:** Department of Physical Geography and Geoinformatics, Faculty of Natural Sciences, Comenius University, Mlynská dolina, Ilkovičova 6, 842 15, Bratislava 4, Slovakia

**Keywords:** Deforestation, Land cover change, Forest monitoring, Landsat, Sentinel 2

## Abstract

Global Forest Change (GFC) provides a widely accessible tool for monitoring tree cover loss and is frequently used by both professionals and the public in discussions of forest dynamics. However, its reliability in specific regions has not been sufficiently evaluated. We assessed its accuracy in part of Slovakia through visual interpretation of Sentinel 2 imagery conducted independently by three operators. The GFC dataset achieved 95% producer’s accuracy and 78% user’s accuracy in detecting tree cover loss. Therefore, it could slightly overestimate loss in the study area, especially in small patches and edge pixels, as the marginality of pixels explained a large part of the disagreement (AUC = 0.76). However, we could also underestimate the loss during its visual identification, as it proved more challenging than anticipated (average agreement between operators was 85%). To complement this analysis, we compared GFC tree cover loss with official forestry logging records across Slovakia. Both datasets reported similar total areas of loss and largely consistent spatial patterns, with a median difference of 6% in their ratio within forestry mapping units. Nonetheless, we identified regions where GFC reported substantially more loss, potentially reflecting errors in forestry records, as well as regions where forestry records indicated more loss, possibly due to temporal misclassification. Overall, the GFC dataset represents an appropriate source for monitoring tree cover loss in Slovakia, provided that users are aware of its limitations.

## Introduction

Tree cover loss within forests is one of the most discussed land cover changes globally, due to its environmental and socio-economic impacts. It contributes to climate change by releasing stored carbon dioxide, and forests thus play an important role in carbon accounting under the Kyoto Protocol and REDD initiatives (Grassi et al., 2017), but it may also lead to increased erosion (Malvar et al., 2017), loss of biodiversity (Nováková & Edwards-Jonášová, 2015; Thorn et al., 2017) and ecological stability (Larsen, 1995). It usually negatively affects the recreational and tourist potential of regions, but simultaneously, it is still an important source of income in many areas; whether directly from timber export (Březina et al., 2024) or indirectly from agricultural use of the cleared lands (Pendrill et al., 2022). In Slovakia, as well as other highly forested countries, discussion about forest management approaches therefore spilt from professional and stakeholder circles into the wider public. Spatial data on tree cover loss thus got into the spotlight. There is a fundamental distinction between deforestation and forest tree cover loss. Deforestation represents a permanent land use change, whereas forest tree cover loss manifests a temporal change of land cover with the land use remaining the same. Only 27% of Global Forest Change in 2001–2015 is attributed to deforestation, while the rest can be attributed to the land cover change caused by forestry (26%), shifting agriculture (24%), and wildfire (23%) (Curtis et al., 2018). In Slovakia, the area of forest parcels has remained relatively unchanged since the 1990 s, accounting for 41.30% of the total area of Slovakia in 2023 (Kapusta, 2023). However, large areas lost their tree cover in this period, not only because of regular harvesting, but also due to windstorms (Falťan et al., 2011), insect infestations (Jakuš et al., 2015), salvage logging (Dobor et al., 2020), and long-term droughts (Jamnická et al., 2024). All these changes utilise the land cover perspective, which will be followed in this paper.

Generally, there are two types of widely available data on forest tree cover loss: forestry records and remote sensing imagery. Employment of remote sensing data inherently frames observed tree cover loss as a land cover change, while empirically assembled forestry records based on field measurements provide all the required information for the exact derivation of tree cover loss extent. Nevertheless, both have their shortcomings. The estimates of the official annual forest-harvest statistics are usually provided at a somewhat coarse spatial scale, and they may be incomplete or irregularly updated in some cases (Ceccherini et al., 2020). In remote sensing, aerial imagery usually does not offer high temporal resolution (Guimarães et al., 2020) and satellite-based identification struggles with its accuracy, mostly due to the consequences of its spatial resolution (Souza et al., 2013) and weak distinction between forests and non-forest vegetation (Fergusson et al., 2020; Hamilton & Casey, 2016).

The simplicity of data acquisition is another crucial aspect, as many applications require ready-to-use spatial data on tree cover loss. A few data sources offer relatively easily accessible country-wide spatial data on tree cover loss in Slovakia: CORINE Land Cover, a component of the European project Copernicus, focuses on monitoring land cover changes since 1990 with a spatial resolution of 100 m, a temporal resolution of 6 years, and an overall accuracy of 87%, 84%, and 92% in 2000, 2012, and 2018, respectively (Büttner et al., 2021). It offers easily accessible raster and vector data whose parameters may be sufficient for some applications, but there is usually a demand for higher temporal and spatial detail of tree cover loss data.

The Global Forest Change (GFC) dataset specifically addresses the presence of annual tree cover loss and also offers higher spatial resolution. Using the processing methods introduced by Hansen et al. (2013), this dataset provides worldwide tree cover loss raster layers based on the classification of 30-m resolution Landsat imagery from 2000 onwards. All vegetation higher than 5 m is considered tree cover, while a stand-replacing disturbance (decrease of tree cover under 50%) is referred to as tree cover loss. GFC poses a valuable data source, especially in terms of its temporal resolution. Due to its simplicity of use, it became a popular source for monitoring tree cover loss. This also increased the need for assessment of its accuracy and limitations when used in specific regions.

Linke et al. (2017) mention that the original GFC data indicate 94% producer’s accuracy (PA) and 88% user’s accuracy (UA) for the temperate and the boreal biomes, but their own validation found lower accuracies in the temperate forest region of Atlantic Canada (PA 82%, UA 81%). Ceccherini et al. (2020) found a similar 82% accuracy on patches larger than 4.50 ha in the European Union, as well as Ramirez-Reyes et al. (2018) found 83% in tropical Mexico (although only 62% in its subtropical regions).

This accuracy tends to be affected by the size of tree cover loss patches: In the Linke et al. (2017) study, the probability that GFC detects tree cover loss was around 90% or greater for patches larger than 7 ha, it gradually decreased to 76% for blocks between 2 and 3 ha, and steeply declined to 59% for the numerous small blocks between 1 and 2 ha (Linke et al., 2017). The accuracy falls even more in studies focusing on regions with dominance of harvesting in smaller patches: GFC detected 45% of slash-and-burn agriculture in Madagascar, but only 19% of selective logging (Burivalova et al., 2015). Only 11% of the forest-loss polygons visually identified on aerial imagery were successfully identified by the GFC dataset in the case study in southern Japan (Yamada et al., 2020). This inability of GFC to capture small areas of disturbances was also identified by Rossi et al. (2019) and Tyukavina et al. (2013), while the classification of mixed pixels was mentioned as one of the most challenging problems: Pixels encompassing heterogeneous land cover (e.g. a mixture of green canopy and bare soil) record mixed spectral signals that are not representative of either class, thereby complicating classification. The prevalence of such mixed pixels increases when patches of tree cover loss are relatively small or exhibit complex geometries – generally, when the proportion of edge pixels relative to the total pixels representing the patch is high. We did not find studies directly assessing the effect of this marginality of pixels on accuracy.

Another issue often mentioned in the studies is the use of imagery obtained mostly during late summer, which can lead to inaccuracies in assigning tree cover loss to a specific calendar year – temporal misclassification. This tends to be problematic, especially in terms of reliable reconstruction of tree cover loss temporal dynamics, which is crucial, particularly in the cases where analyses incorporate information from different datasets.

In general, these inaccuracies typically result in an underestimation of the GFC tree cover loss area compared to validation data, as found in several studies from the period 2000 to 2012, according to Vancutsem et al. (2021). On the other hand, GFC overestimated tree cover loss by 55–100% in the case study of Kinnebrew et al. (2022) in Amazonia. A general trend of over- or underestimation is therefore rather unclear.

We did not find any study validating GFC data specifically for Slovakia; therefore, the 82% accuracy identified by Ceccherini et al. (2020) appears to be the closest available estimate; however, these results were questioned by other authors (Breidenbach et al., 2022; Palahí et al., 2021; Wernick et al., 2021). The study found an increase in harvested area in the EU between 2004–2015 and 2016–2018 (51% for Slovakia), although after the criticism, they revised the estimate to 30% (Grassi et al., 2021). Similarly, Turubanova et al. (2023) identified an increase in annual tree canopy removal in Europe since 2013. These interpretations on temporal dynamics and other aspects of tree cover loss have serious implications for the discussion on forest management and are therefore a good example of why a critical evaluation of the GFC dataset is important.

Alternative data for European countries can be accessed through the European Forest Disturbance Map, introduced by Senf and Seidl (2021). An additional repository contains attributes of each disturbance patch to bark beetle/windstorm, fire, and other disturbances (e.g., salvage logging). Just like GFC, the computation of these datasets is based on the classification of 30-m resolution Landsat imagery. Information about tree cover loss in European countries can also be obtained from 20-m resolution Tree Cover Presence Change layers, which are released every three years by the Copernicus Land Monitoring Service.

Forestry records are another source of fine-scale data on tree cover loss. In Slovakia, several forestry datasets are available containing disturbance-related information at the national level. The National Forestry Centre provides vector layers of basic forest management units (JPRL), as well as corresponding annual records of logging. Numerous attributes are included in each record, including disturbing factor, type of logging execution, logged area, and volume of removed wood (or unextracted deadwood) for each tree species. We did not find any studies evaluating the accuracy of these logging records.

Satellite-imagery-based and forestry logging records provide us with a comprehensive overview of tree cover loss while using different sources, methodologies, and formats of their spatial representation. Given that this is a topic of significant environmental and socio-economic relevance, we believe that it is necessary to understand differences in their depiction of tree cover loss in countries like Slovakia. There is also a gap in information about the accuracy and over/underestimation of GFC data in Central Europe, which, due to its ease of access, spatial uniformity, and timeliness, is increasingly being used as a reference for information regarding tree cover loss.

Therefore, this study aims to evaluate the accuracy of the GFC tree cover loss data in Slovakia. We intend to assess the accuracy of the detected tree cover loss through the visual interpretation of Sentinel 2 imagery carried out by three independent operators. We also aim to compare the GFC data with the forestry logging records. Lastly, we try to explain disagreements with a special focus on the effects of data resolution and geometry.

## Methods

### Study area

The comparison of GFC and forestry records includes the entire area of Slovakia, while the visual accuracy assessment of GFC was conducted within the extent of the Sentinel-2 tile R036/T34UDV, which covers a large part of northern and central.

**Fig. 1:**
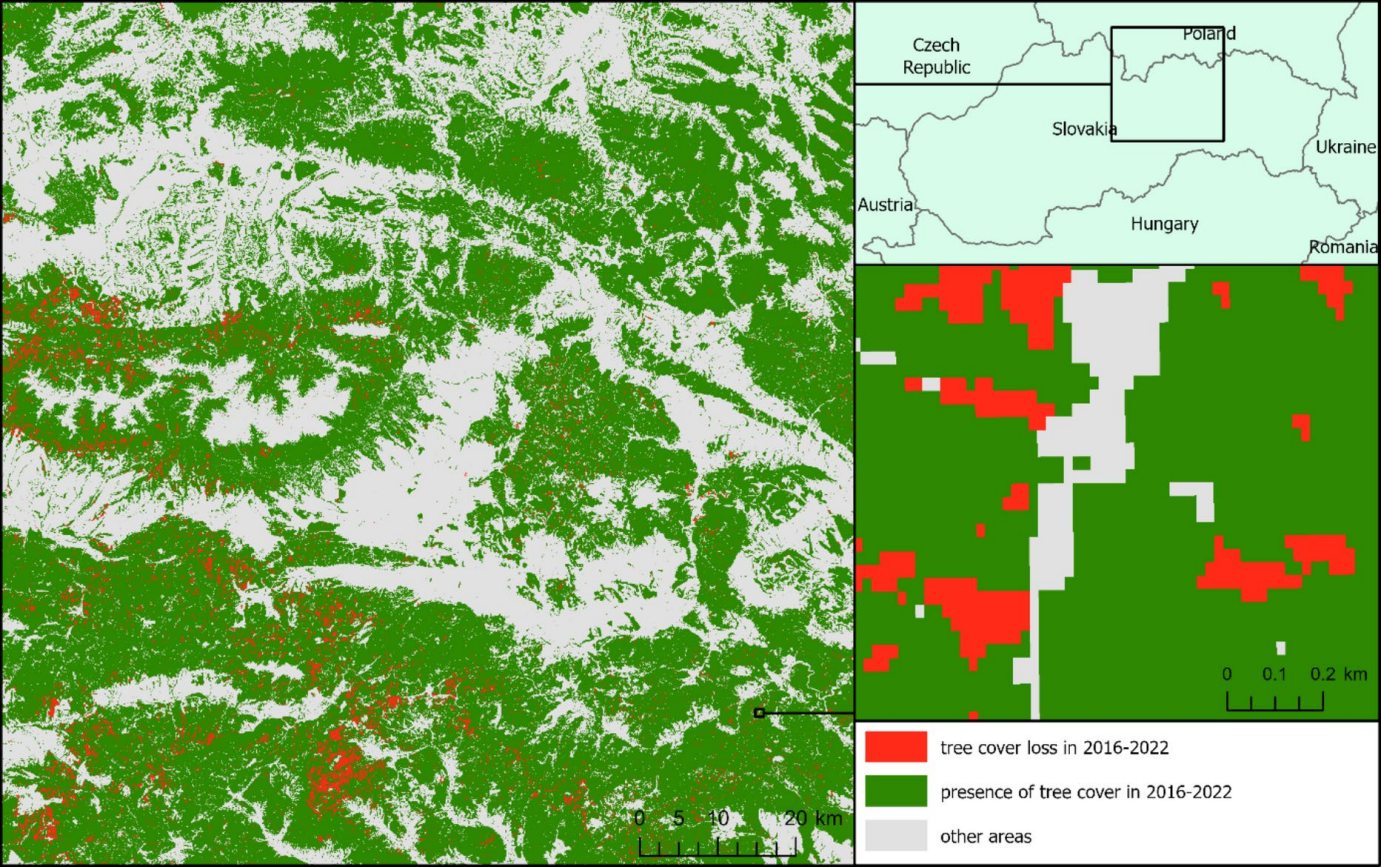
Tree cover loss in 2016–2022 according to GFC in the study area (source: Hansen et al., 2013)

Slovakia (Fig. 1). This area encompasses several mountain ranges and forested landscapes, including the Tatras, Low Tatras, the northern part of Slovenské rudohorie, Branisko, Čergov, Levočské vrchy, Spišská Magura, and Pieniny, as well as parts of the Orava region. These subregions represent some of the most dynamic forest environments in Slovakia, strongly shaped by windstorms, bark beetle outbreaks, and intensive logging.

### GFC data

Tree cover loss, as reported by GFC, results from the classification of Landsat multispectral data. This raster is provided in the geographic coordinate system WGS 1984 (EPSG 4326), with a cell size of 0.00025 degrees—this translates to a rectangle measuring approximately 27.50 m × 18 m in northern Slovakia when projected in S-JTSK (EPSG 5514). The raster indicates the year in which the tree cover loss was first detected by satellite imagery, spanning from 2000 to the present (Hansen et al., 2013). Due to imagery acquisition dates, the forest tree cover loss which physically happened at the end of one year is detected in the following year. To mitigate this issue, we opted to assess the accuracy of the tree cover loss over an extended timeframe—we used a layer depicting tree cover loss for the years 2016–2022 (Fig. 1). Tree cover loss detected in any of the specified years was marked as *loss*, presence of tree cover throughout the entire period as *noloss*, and absence of tree cover at the beginning of the period was excluded from the analysis as *other areas*.

### Visual accuracy assessment of GFC data

We evaluated the accuracy of GFC tree cover loss utilising Sentinel 2 multispectral data (European Space Agency, 2024). Similarly to the GFC data, it is distributed in the geographic coordinate system WGS 1984, but with a grid size of 0.000112 degrees. It corresponds to a rectangle of roughly 12.5 m × 8.3 m in the projected coordinate system S-JTSK in the Tatra region. Consequently, there are around 4.8 pixels of Sentinel 2 data for each pixel of GFC data.

**Fig. 2:**
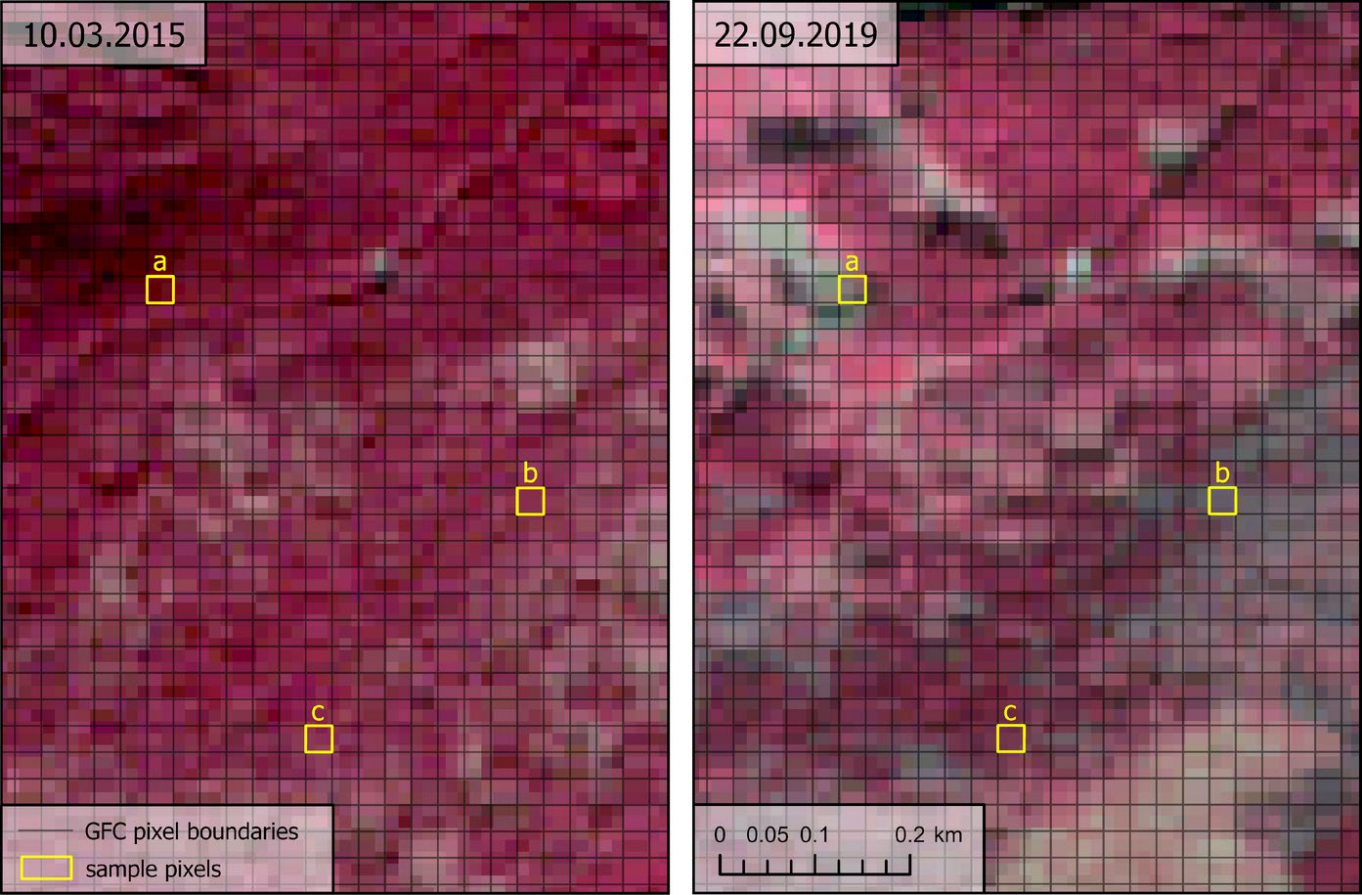
Comparison of the spatial resolution of Sentinel 2 imagery (false IR image) and GFC data (outlines of pixels) with examples of pixels with *noloss* and *loss* due to cutting and drying of the forest. Sample pixels identified as a) *loss* – cutting, b) *loss* – drying, and c) *noloss* (source: European Space Agency, 2024; Hansen et al., 2013)

The assessment was based on a stratified random sample from the GFC raster pixels comprising 1000 randomly selected *loss* pixels and 1000 randomly selected *noloss* pixels. Sampling was restricted to GFC pixels overlapping with the Sentinel 2 data tile “R036_T34UDV” (Fig. 1) within Slovakia. All analyses were performed in ArcGIS Pro. A “blinded” layer was generated from the sample, where the sample pixels were converted to their polygon outlines, randomly arranged in the table, and the GFC tree cover loss data were removed. The outlines of each pixel were then displayed over the Sentinel 2 imagery (Fig. 2) using level 2 A data, which include bottom-of-atmosphere corrections. A visual identification of the presence or absence of tree cover loss (as defined by Hansen et al., 2013) within each pixel was conducted by analysis and comparison of the RGB and false IR images from various dates:

- Initial image: 3.10.2015 (auxiliary image: 8.8.2016)
- Auxiliary mid-period image: 22.9.2019
- Final image: 25.12.2022 (auxiliary images: 3.7.2022, 3.6.2023)

Three categories were recognised in the visual accuracy assessment: *No tree cover loss* (0) was identified when tree cover persisted over 50% of the GFC sample pixel during the entire period. *Tree cover loss* (1) was identified if any reduction in tree cover falling below 50% of the pixel occurred—this encompassed clear-cut, selective logging of green and/or dry trees, as well as manifestations of drying of the forest (whether temporal or permanent). These rules were adopted to replicate the methodology of the GFC dataset (Hansen et al., 2013). The identification of tree cover loss was limited by the spatial resolution of Sentinel 2 imagery (Fig. 2). We therefore visually identified the land cover of the broader area surrounding the GFC sample pixel to interpret which colours of Sentinel 2 pixels represent which land cover. We then assessed the ratio of Sentinel 2 pixels with various interpretations within the GFC sample pixels and estimated the ratio of tree cover. The decline in tree cover below 50% could be clearly identified in one image or contextually interpreted on images from several dates. We separately identified cases when the *tree cover* was already *below 50% in the initial image* (2). The identification was carried out using the geographic coordinate system WGS 1984, which is native to both Landsat and Sentinel 2 satellite data.

To enhance the reliability of the accuracy assessment, three independent operators performed this visual identification of tree cover loss. Then their results were compared. We determined the *overall agreement* (ratio of identically identified pixels to all pixels) and three confusion matrices for comparison of all three pairs of operators. From them, we calculated: the *mean overall agreement of pairs*—a ratio of pixels identified identically by both operators to all pixels, averaged for all pairs; and the *mean agreement on categories*—an average of “producers” and “users” accuracies for each category (0, 1, 2) from all confusion matrices.

The overall result of the visual accuracy assessment was defined principally as the mode of the results of operators: *loss* if at least two operators identified “1” (tree cover loss) and *noloss* if at least two operators identified “0” (tree cover without loss), or one identified “0” and one “2” (did not find sufficient tree cover in the initial images). If at least two operators identified “2”, we excluded the pixel from further analysis. The overall result was joined with the original sample data and compared with the GFC data in a confusion matrix.

### Effect of marginality of GFC loss pixels

**Fig. 3:**
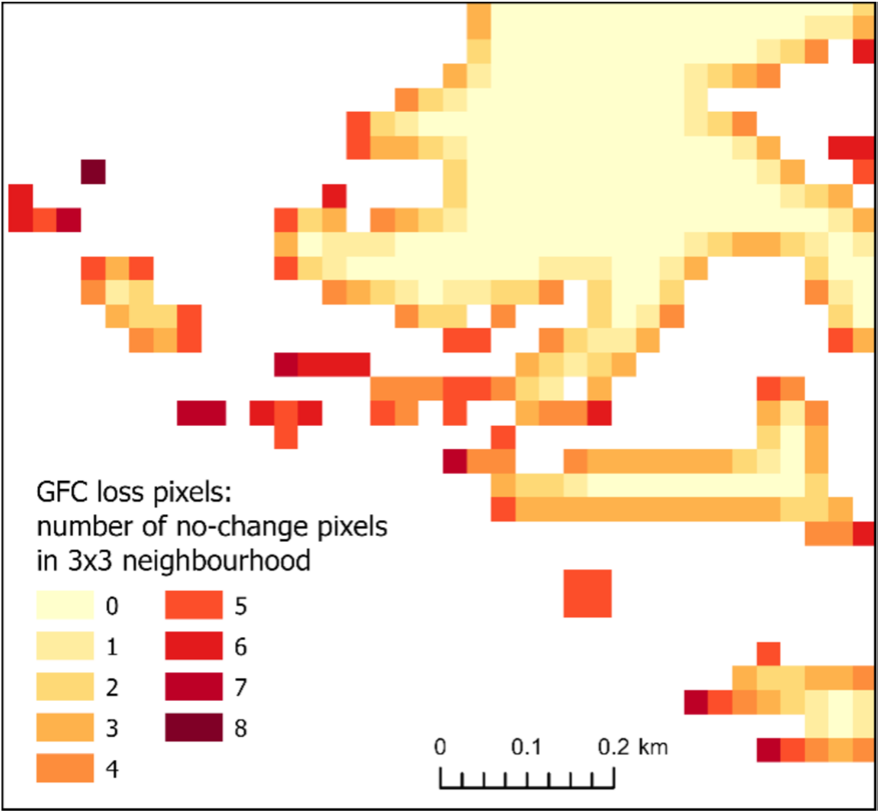
An example of the marginality index of GFC tree cover *loss* pixels – the number of no-change pixels in a 3 × 3 neighbourhood. (source: derived from Hansen et al., 2013)

To evaluate the extent to which mixed spectral signal at the edges of tree cover loss patches could explain disagreement between GFC data and our visual identification, we quantified the *marginality* of the pixels within GFC *loss* patches: For each *loss* pixel, we calculated the number of no-change pixels within its 3 × 3 neighbourhood (Fig. 3), when no-change represent all pixels other than *loss* pixels (including GFC *noloss* and pixels with other land cover). Marginality index 8 thus represents a lone *loss* pixel, value 4 is typically a pixel at the straight boundary of the *loss* patch, and 0 means a pixel completely inside the *loss* patch. Higher marginality means a higher probability of the problem with mixed signal and therefore a hypothesised higher uncertainty of the classification. We used the same principle to quantify the marginality of pixels within GFC *noloss* patches.

To visually assess this effect, histograms of the marginality were created for the following: (1) GFC *loss* pixels; (2) GFC *noloss* pixels; (3) GFC *loss* pixels where our visual identification confirmed *loss*; (4) GFC *loss* pixels where we visually identified *noloss*; (5) GFC *noloss* pixels where we identified *noloss*; and (6) GFC *noloss* pixels where we identified *loss*. To quantify this effect, we also calculated the Area Under the Curve (AUC) of a logit regression model, where the marginality was used as the independent variable. The first model assessed the power of the marginality index to discriminate between GFC *loss* pixels, where we identified *loss* and *noloss*; the second model was applied analogously for GFC *noloss* pixels.

### Comparison of GFC tree cover loss and forestry logging records

We performed this comparison on the entire area of Slovakia in the period 2019–2023 (due to data availability). We used the forest compartment data freely provided by the National Forest Centre of Slovakia. Data on the logging area for the years 2019–2023 were obtained in tabular form for each year from *data.slovensko.sk* (National Open Data Catalogue, 2024). Records are held for each forest management unit “JPRL” and each tree species in it; therefore, we summarised the logging areas of all species into one value for each JPRL. This data was then joined with JPRL polygons (National Open Data Catalogue, 2024) based on their unique “IDPS” codes—the corresponding JPRL layer was used for each year. We then calculated the percentual share of the logging area on the total area of JPRL for each year (calculated in S-JTSK), overlaid all years into one layer using the Union tool, and summed the logging area shares. The final layer thus consisted of 694,669 polygons with information on the percentual share of the area of logging in years 2019–2023, according to the forestry records.

**Fig. 4:**
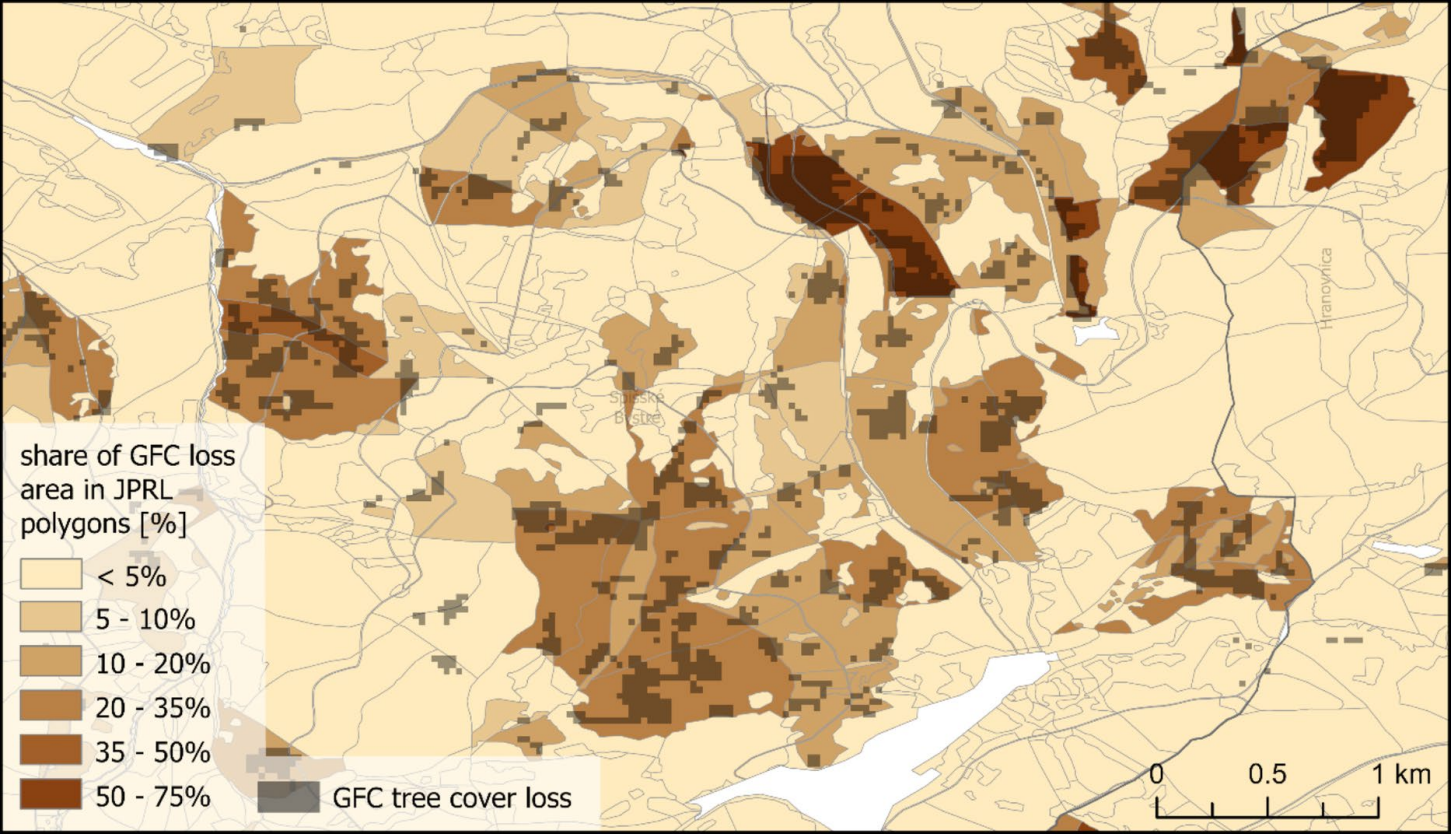
Overlay of the GFC tree cover loss in 2019–2023 raster and forestry records JPRL polygons with the share of logging area (source: Hansen et al., 2013; National Open Data Catalogue, 2024)

For the same JPRL polygons, we determined the percentual share of overlapping pixels of the GFC tree cover loss in corresponding years (Fig. 4) by the Zonal Statistics tool (in WGS 1984 to match the GFC data). During this, a pixel size of 0.00005 degrees was used for the JPRL polygons rasterisation to preserve sufficient detail—it is considerably finer than the resolution of the GFC data (0.00025 degrees).

Thus, we obtained information on tree cover loss in 2019–2023 for each JPRL polygon based on both forestry logging records and GFC data, which we then compared. For their statistical comparisons, we used only those JPRL polygons where a non-zero loss was identified by either GFC or forestry data. This subset comprised less than one tenth (44,911) of the total polygons. We used this subset to focus on areas of tree cover loss, since comparing areas with zero loss in both sources would be uninformative. In the statistical assessments, polygons were weighted by their area.

## Results

### Agreement among operators

The share of sample pixels, in which all operators agreed on classification (Table 1), was 77.50% (Table 2). When comparing results between two operators, the mean agreement of these pairs was 84.80%. We also assessed agreement in the pairs for distinct classes to estimate the difficulty of their visual identification on Sentinel 2 images: The highest mean accuracy was found in the identification of persistent tree canopy (88.50% – category 0), followed by tree cover loss (84.80% – category 1). The lowest agreement was achieved in category 2—areas already without tree cover at the beginning of the study period (65.20%).

**Table 1:** Agreement in pairs of operators on visual identification of tree cover loss

A-B		Operator A			*acc. A to B*	A-C		Operator A			*acc. A to C*	B-C		Operator B			*acc. B to C*
		0	1	2				0	1	2				0	1	2	
Operator B	0	952	93	67	*86%*	operator C	0	909	71	55	*88%*	Operator C	0	952	69	14	*92%*
	1	72	661	45	*85%*		1	100	651	17	*85%*		1	120	643	5	*84%*
	2	10	7	93	*85%*		2	25	39	133	*68%*		2	40	66	91	*46%*
*acc. B to A*		*92%*	*87%*	*45%*	***85%***	*acc. C to A*		*88%*	*86%*	*65%*	***85%***	*acc. C to B*		*86%*	*83%*	*83%*	***84%***

**Table 2:** Overall agreement of operators on visual identification of tree cover loss

Overall agreement:	77.50%
Mean overall agreement of pairs:	84.80%
Mean accuracy of category 0:	88.50%
Mean accuracy of category 1:	84.80%
Mean accuracy of category 2:	65.20%

### Accuracy of GFC data

**Fig. 5:**
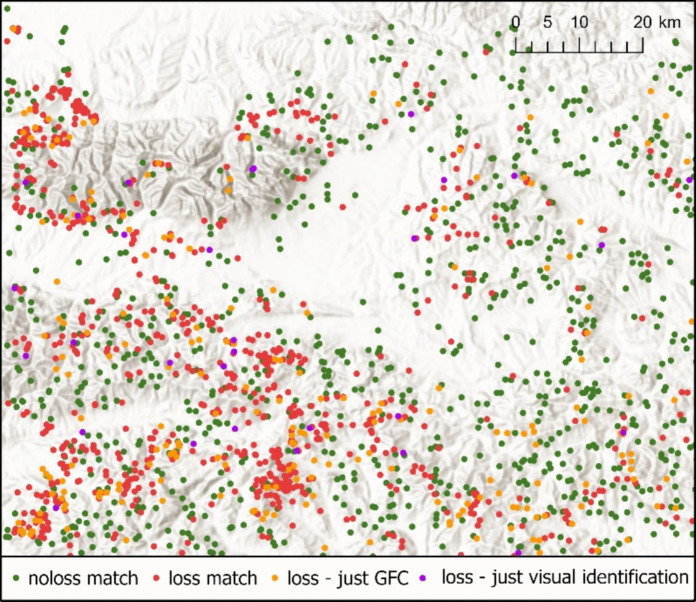
Comparison of tree cover loss in 2016–2022 according to GFC and visual identification

The overall agreement of the GFC data with the result of our visual identification (Fig. 5) is 86.90% (Table 3). We found high producer’s accuracy and relatively lower user’s accuracy of the GFC tree cover loss layer: GFC detects 96.10% of visually identified tree cover loss, while we visually identified just 77.60% of the GFC tree cover loss pixels. Therefore, GFC overestimates the tree cover loss compared to our data by 24% (944 GFC identified to 763 visually identified pixels). However, this difference can be partially explained by the marginality of the GFC loss pixels.

**Table 3:** Accuracy of GFC data assessed by visual interpretation

		GFC		Producer’s accuracy
		0	1	
Visual accuracy assessment	0	869	211	*80.50%*
	1	30	733	***96.10%***
User’s accuracy		*96.70% 96.70%*	***77.60%***	***86.90%***

### Effect of marginality of GFC loss pixels

**Fig. 6:**
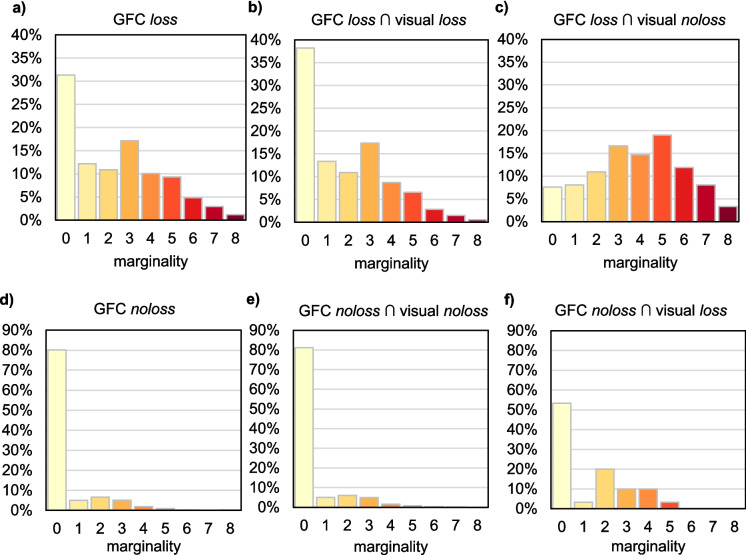
Marginality of sample pixels of GFC *loss* (**a**) and *noloss* (**d**) with different outcomes of our visual accuracy assessment: GFC *loss* pixels where we did not visually identify *loss* (**c**) show higher marginality than those where the *loss* was confirmed (**b**). GFC *noloss* pixels show a similar effect (**e**, **f**), although it is less apparent due to a generally smaller share of marginal *noloss* pixels (**d**)

The areas of GFC tree cover *loss* are considerably fragmented, as shown by the histogram of their marginality in our area (Fig. 6a). The average marginality value is 2.32, indicating that a typical *loss* pixel is adjacent to 2–3 pixels of other land cover. About 45% of GFC loss pixels have more than three such neighbours, increasing the probability of a mixed signal.

However, when we split the GFC loss into subsamples based on whether the loss was visually confirmed, the histograms differ: Pixels with confirmed *loss* show a similar distribution with a slightly lower average (1.87; Fig. 6b), while pixels without confirmed *loss* have considerably more neighbours with other land cover, with an average of 3.87 and a different histogram (Fig. 6c). Therefore, disagreement between the visual and GFC detection of tree cover loss typically occurred on marginal pixels of GFC *loss* areas. This effect of marginality can explain about half of the disagreement between visual and GFC *loss* detection, according to the logistic regression model (AUC = 0.76).

The areas of GFC *noloss* are much less fragmented (Fig. 6d), with average marginality value of 0.48, but the effect of marginality is similar: Pixels with visually confirmed *noloss* show comparable histogram (Fig. 6e) and an average marginality of 0.44, whereas pixels with visually identified *loss* include more cases with marginality values of 2–5 (Fig. 6f) and have a higher average of 1.30. According to the logistic regression model, marginality was able to explain a relatively smaller part of the disagreement between visual and GFC *noloss* detection (AUC = 0.65).

### Comparison of GFC tree cover loss and forestry logging records

**Fig. 7:**
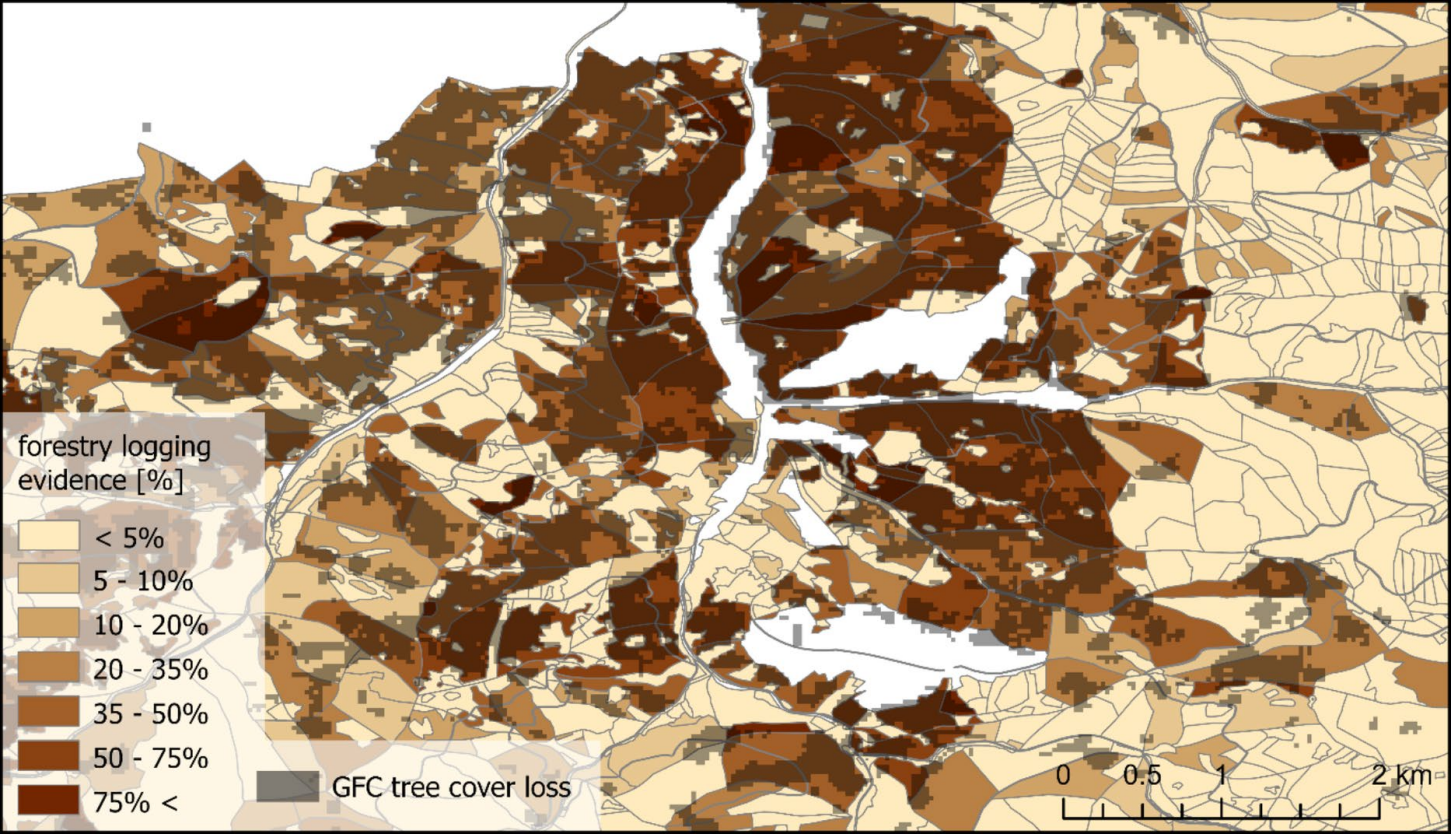
Tree cover loss in 2019–2023 in the Čierny Balog area according to the forestry records and GFC (derived from National Open Data Catalogue, 2024 and Hansen et al., 2013)

GFC tree cover loss and polygons of forestry logging records generally show similar areas. The main differences arise from different technical forms of data. The raster form of GFC data shows information evenly, but it is restricted to the resolution of Landsat (27.50 m × 18 m in this region), and the information is binary. The forestry records carry information unevenly: In some areas, the vector polygons offer higher resolution than Landsat (e.g. boundaries of forest roads, some tiny or branched polygons). But most areas are covered by large JPRL polygons with information about logging area share, which do not allow locating the specific areas of logging. Therefore, in general, the GFC data offer a more explicit localisation of tree cover loss (Fig. 7).

**Fig. 8:**
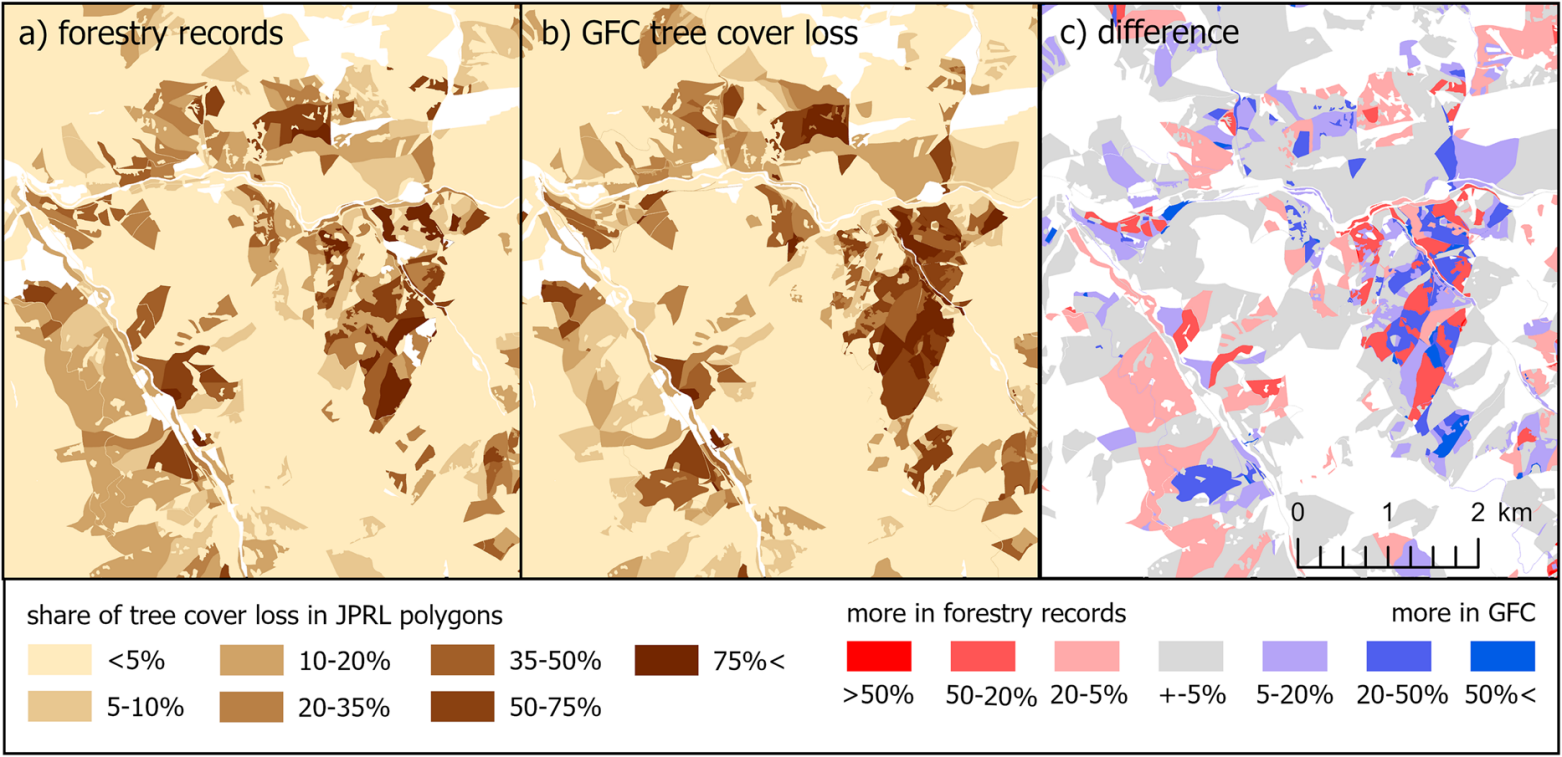
Comparison of the share of tree cover loss area in forest management units (JPRL) according to the a) forestry records, b) overlayed GFC tree cover loss, and c) their difference (derived from National Open Data Catalogue, 2024 and Hansen et al., 2013)

The differences in the forestry records logging share and the share of GFC tree cover loss area in forest management unit (JPRL) polygons are depicted in an example in Fig. 8. In areas like these, polygons with surpluses in GFC data tend to neighbour polygons with surpluses in forestry records. In some cases, forestry records even report logged areas larger than the polygon itself. These findings suggest errors in the records, likely due to difficulties in boundary identification during logging: many JPRL polygons are small and irregularly shaped, which may have led to logging being registered to neighbouring polygons.

**Fig. 9:**
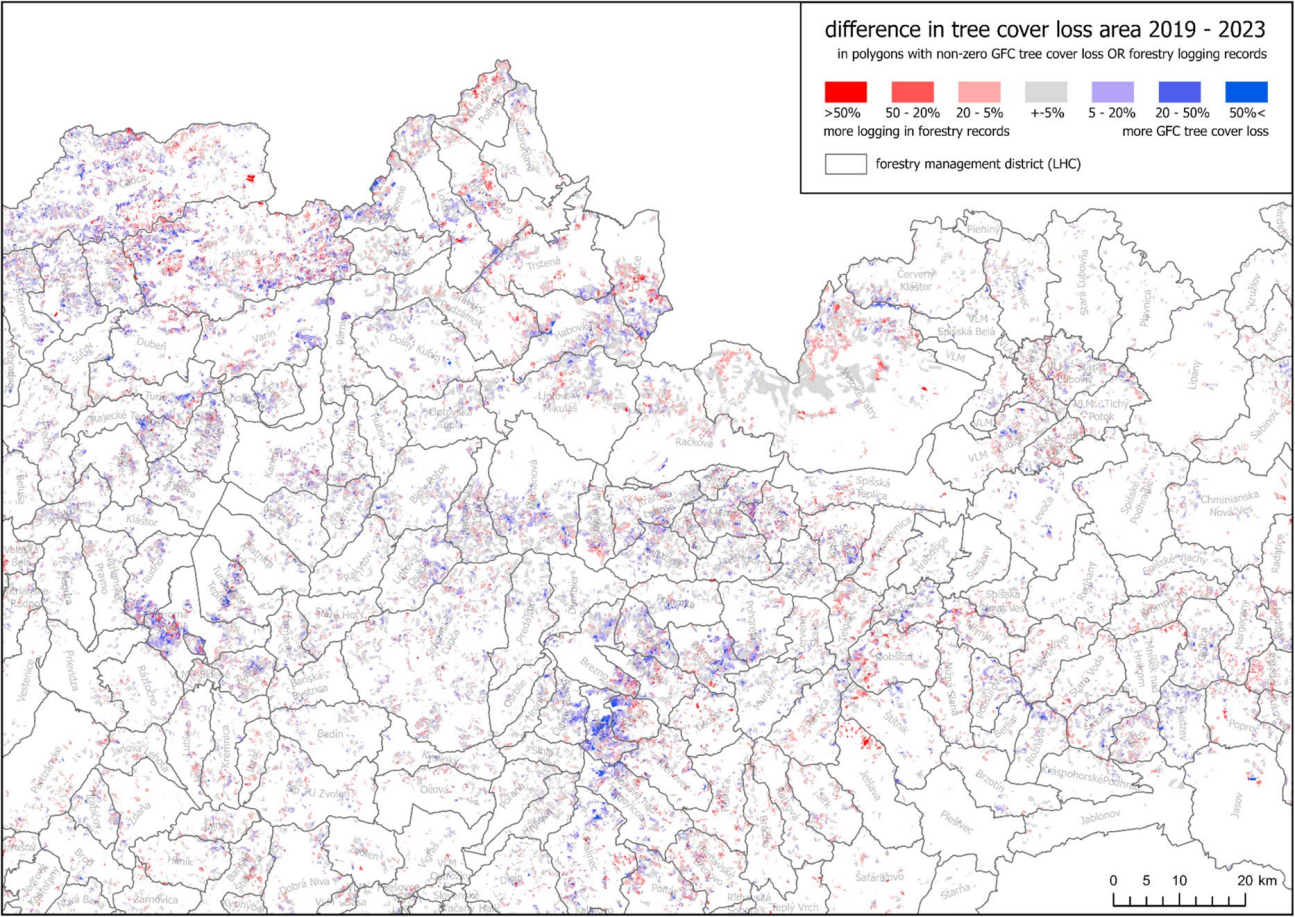
Difference in the share of tree cover loss area in forest management units (JPRL) between forestry logging records and GFC in 2019–2023 in part of central and eastern Slovakia (derived from National Open Data Catalogue, 2024 and Hansen et al., 2013)

However, there are numerous regions where one or another difference dominates (Fig. 9). Significantly more GFC tree cover loss was detected mostly in Čierny Balog region, for example, in the forestry management districts (LHCs) Dobroč and Šaling, and the south of Turiec region (e.g. Horná Štubňa and Turčianske Teplice). On the contrary, more loss according to forestry data was recorded e.g. in the central Gemer region (e.g. LHCs Jelšava and Dobšiná) and some units in eastern Slovakia (e.g. LHCs Opátka, VLM Jovsa). There are also forestry management districts with both differences being high, especially in northern Slovakia (e.g. LHCs Čadca, Krásno, Oravice).

**Fig. 10:**
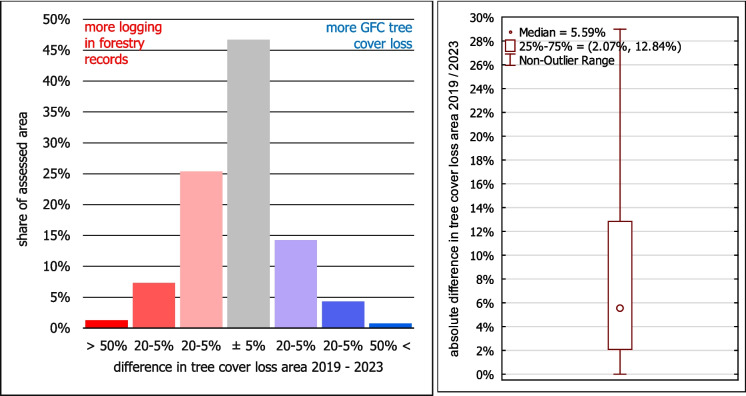
Left: Differences in the share of tree cover loss area in JPRL polygons between forestry logging records and GFC in 2019–2023 in the entire Slovakia; Right: Absolute values of these differences

The absolute values of the differences between the GFC and the forestry records (Fig. 10, right) show their relatively good fit. The median absolute difference is 5.60%, and the upper quartile is 13% – three-quarters of the polygons’ area *with registered logging OR tree cover loss* has a difference lower than 13%. We believe that a substantial part of these differences can be attributed to the aforementioned variations in methods and technical forms of spatial representation.

The histogram of the differences (Fig. 10 left) shows a slight shift towards red—the logging according to the forestry records is not captured by GFC a bit more often than vice versa. However, the total area of logging in 2019–2023 according to the forestry records (10,522 ha) is surprisingly similar to the total area of GFC tree cover loss (10,586 ha)—their difference is just 0.60%.

## Discussion

### Visual interpretation

Our 85% average agreement between two operators highlighted that medium-resolution satellite imagery, such as Sentinel 2, has certain limitations in capturing tree cover loss (Havašová et al., 2017; Kinnebrew et al., 2022) and may not reflect minor tree cover reductions (Murgia et al., 2025). Aerial imagery would offer significantly higher spatial resolution, but less suitable temporal resolution, as aerial images are mostly acquired in summer and therefore do not align well with GFC data, which aggregates *loss* by calendar year. We therefore prioritised the temporal resolution to reduce the risk of temporal misclassification.

**Fig. 11:**
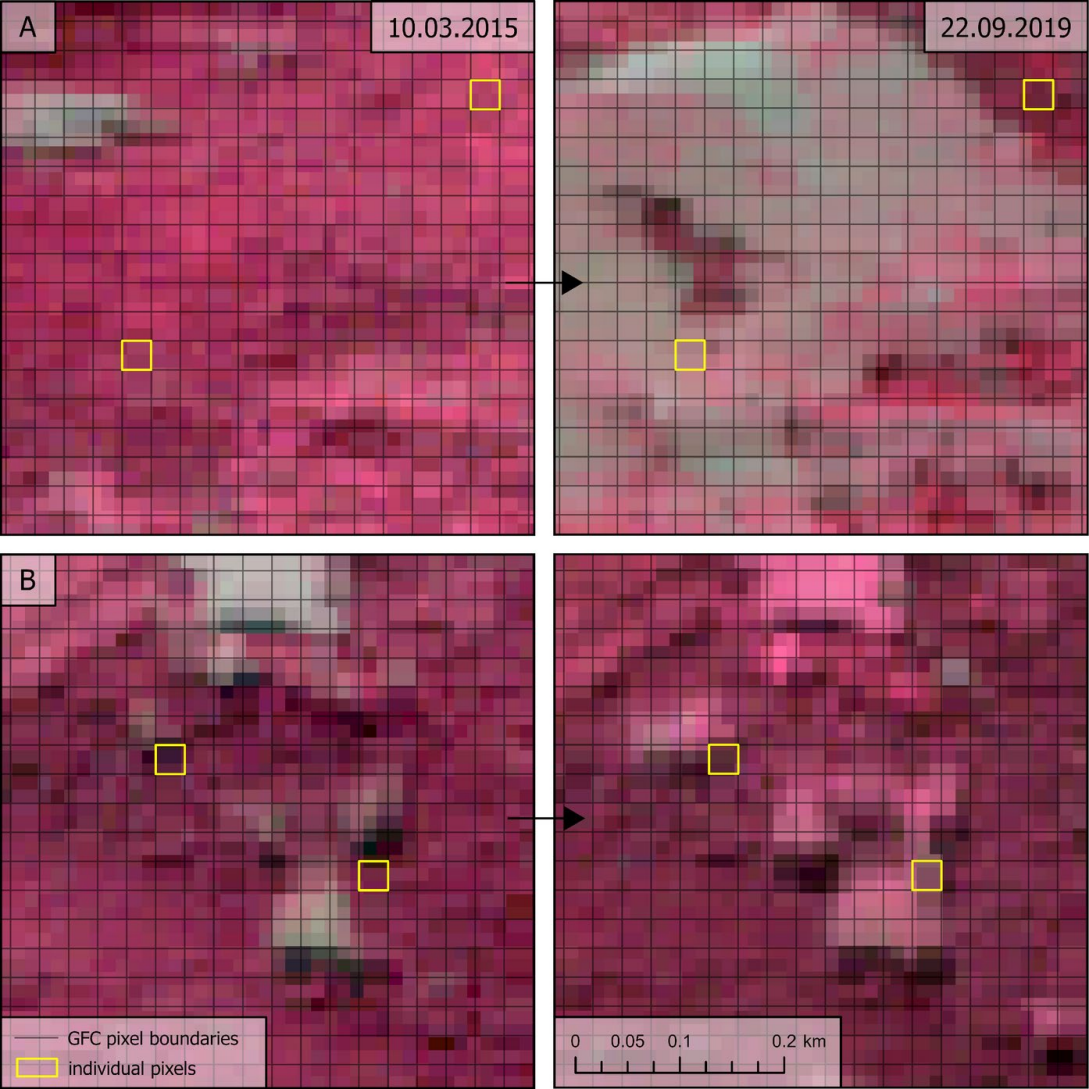
Examples of the pixels with: **A** unambiguous interpretation, **B** challenging interpretation. (source: European Space Agency, 2024; Global Forest Watch, 2024)

Visual identification of *loss* on Sentinel 2 imagery proved challenging in certain sample pixels (examples in Fig. 11). Some disturbances were too small for Sentinel 2 spatial resolution, such as minor logging patches or thinning. In many cases, the forest had low density already in the first image, making it difficult to determine whether tree cover had decreased below the 50% threshold before 2016, during the study period, or at all. The presence of shrubs beneath logged trees posed an additional challenge for detection. However, most sample pixels had an unambiguous interpretation: they either showed a persistent canopy across all images or a clear-cut visible in the 2019 or 2022 imagery (and in some cases already in the 2015–2016 imagery, thus classified as “2”).

The challenges of visually identifying minor loss patches on Sentinel 2 imagery are consistent with the findings of Castillo et al. (2022), who reported an overall accuracy of 83% in classifying selective logging in the Amazon when compared with very-high-resolution UAV imagery, but only 35% accuracy in classifying disturbed canopy. In this light, the GFC tree cover loss data are remarkably accurate, considering that they are based on coarser Landsat imagery and the detection algorithm is trained to work globally.

### Accuracy assessment of GFC

Disagreement between GFC data and our visual interpretation is often localised on the edges of the tree cover *loss* patches, as shown by our marginality analysis (Fig. 6). Previous works outlined that the ability of the GFC dataset to detect tree cover loss declines with the shrinking of the tree cover loss patches’ area (Burivalova et al., 2015; Ceccherini et al., 2020; Linke et al., 2017; Yamada et al., 2020). Our marginality analysis extends and specifies these findings by focusing on the mixed pixels, as they are the direct causal problem complicating the classification of small tree cover loss patches. This problem affects the boundaries of *all* patches; the small ones are more problematic just because a larger share of their area (or even entire area) is captured by the mixed pixels. Therefore, if a significant portion of tree cover loss pixels in the study area is supposed to be captured by mixed pixels, application of methods targeting this problem may be relevant, such as spectral unmixing (Bullock et al., 2020) or OBIA – Object-Based Image Analysis (Navarro Cerrillo et al., 2019).

According to our assessment, GFC data were sensitive (PA: 96%) in capturing tree cover loss visually identified by our operators, but had weaker specificity (UA: 78%). The UA is similar to that identified by Linke et al. (2017) in Atlantic Canada, where they determined that 81% of the GFC tree cover loss was in fact harvested. However, the sensitivity of GFC in our study was notably higher, as only 82% of the total harvested area in the study of Linke et al. was detected by GFC. Results of Yamada et al. (2020) differed even more, with UA of GFC data of approximately 50–80% and PA 0–60% (both increasing with the patch size from < 0.36 ha to 2.25 < ha), but they used significantly finer ground truth data.

Therefore, according to our accuracy assessment, the total area of GFC tree cover loss can be overestimated by 18% in the study area in northern Slovakia. This could be considerably explained by the abundance of pixels with mixed spectral response (edges of the logging patches, selective cut, and thinning, Fig. 6c) – if these pixels were excessively included in the loss layer by the GFC algorithm (the threshold for inclusion was too low), it caused slight spatial inflation of the patches that were otherwise correctly detected. Linke et al. (2017) found a similar effect when 22% of the GFC tree cover loss false positives were actually areas of selective cutting. Considering this uncertainty resulting from the mixed pixels, it would be beneficial if GFC offered a probability layer of the tree cover loss detection, alongside the current binary layer. This would allow users to choose a threshold defining the tree cover loss best suited to their area and the needs of their applications.

A fundamental approach to reducing mixed‑pixel issues is the use of higher‑resolution satellite imagery. In Europe, the Copernicus High‑Resolution Layers provide such an alternative, as shown by Cesaretti et al. (2025) and Labohm et al. (2025), although the design of these datasets differs slightly from GFC: The Tree Cover Presence Change layer is a ready‑to‑use product with 20‑m resolution, but it is released on a three‑year cycle. The Tree Cover Density layer is available annually at 10‑m resolution; however, deriving change from it may not be straightforward. A separate validation of these datasets would be required to determine their accuracy in comparison with GFC.

Part of the disagreement between our results and GFC data could possibly be explained by the temporal accuracy – a problem in assigning tree cover loss events to individual years (Rossi et al., 2019). Imagery from winter months is less suitable and therefore less used for loss detection. Events that occurred during winters 2018–2019 and 2023–2024 thus naturally had a higher probability of temporal misclassification. Linke et al. (2017) found that 85% of the tree cover loss was assigned to the correct year, while nearly 10% was assigned to the next year. Considering that our study period covers just 5 years, this effect may not be negligible. This is also underscored by our results, which showed that the operators had difficulties agreeing on the identification of pixels without tree cover that already existed at the beginning of the study. Temporal misclassification also affects our comparison of GFC with the forestry logging records and can therefore explain a substantial part of their differences.

### Comparison of GFC and forestry records

GFC and forestry logging records are both freely available in Slovakia, but while the GFC data are easily visualised on the internet or downloaded for desktop GIS analyses, the forestry records require some effort to join with spatial data. GFC is an automated algorithm without human interference (positive or negative), whereas forestry records are human-dependent and carried out by many forestry workers directly on-site, which can be advantageous but can also lead to possible errors.

Regarding the comparison of GFC with the forestry records, both data sources showed a generally similar pattern: The differences tended to be under ± 10% (Fig. 10), and they agreed on the total area of tree cover loss. Similarly, Ceccherini et al. (2020) state a congruence between the total forest area of Slovakia, as referred by GFC and FAO. This contrasts with the overestimation of GFC tree cover loss found by our visual interpretation. This may indicate that our visual interpretation is overly conservative in detecting the tree cover loss, while the GFC detection is actually more accurate. Another validation using imagery with higher spatial resolution would allow clearer conclusions, but the study period would need to align with the (usually limited) temporal resolution of such data.

**Fig. 12:**
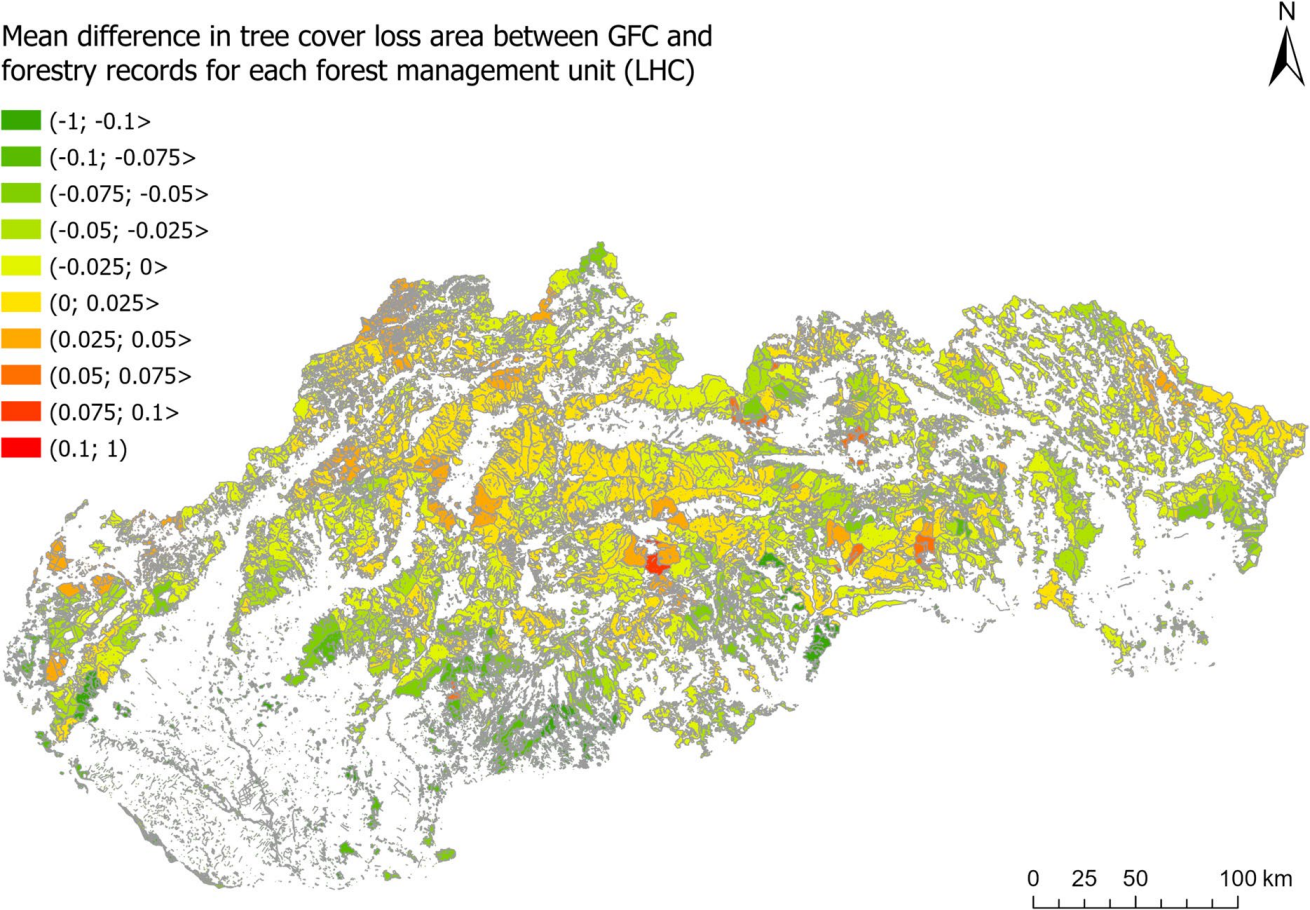
Mean difference in tree cover loss area (2019–2023) between the GFC and the forestry records calculated for each forest management district (LHC) (source: derived from National Open Data Catalogue, 2024 and Hansen et al., 2013)

**Fig. 13:**
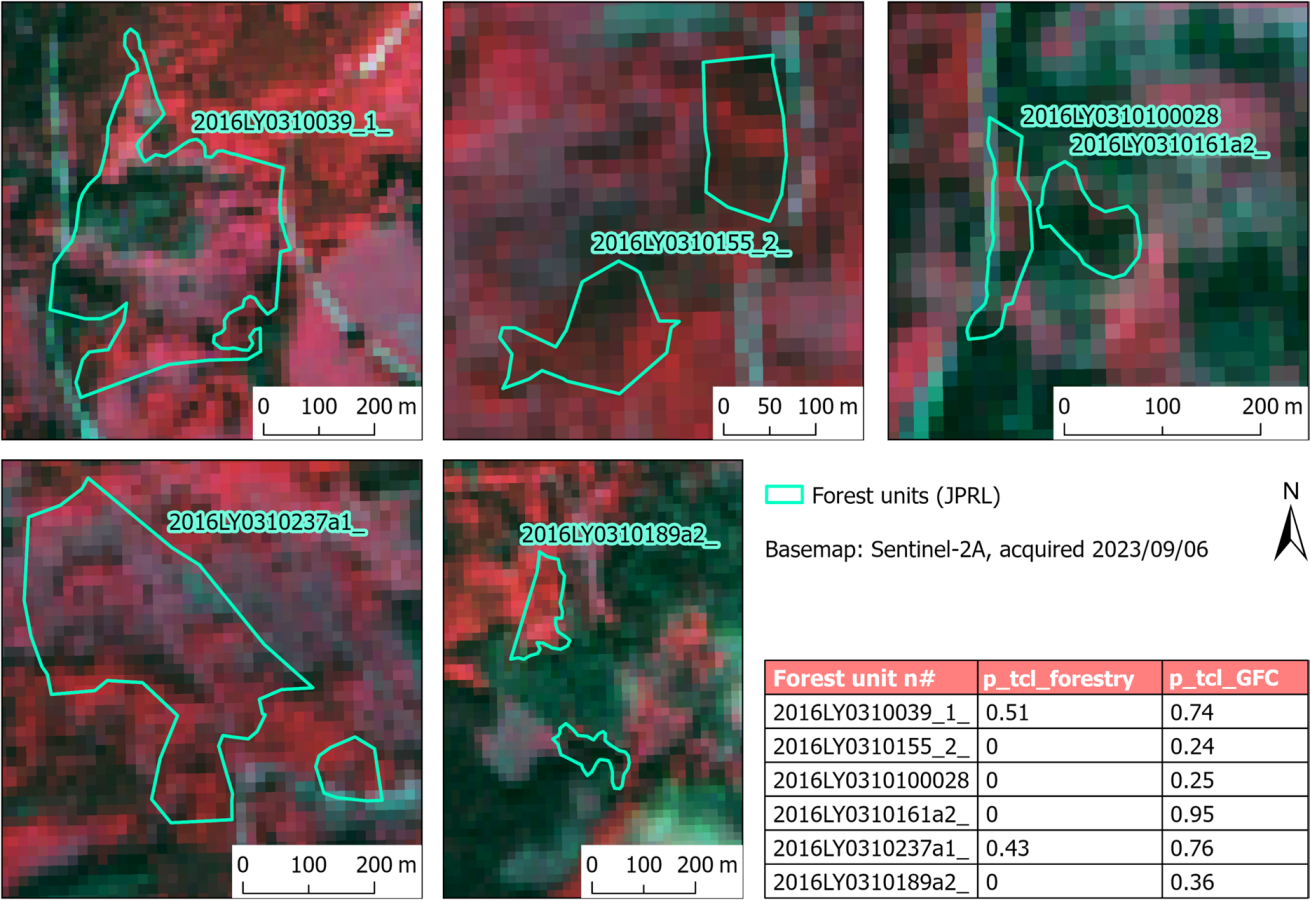
Differences between the actual state (2023) and proclaimed tree cover loss proportion according to the forestry records (p_tcl_forestry) and the GFC (p_tcl_GFC) in the case of six randomly selected forest management units (JPRL) in Dobroč forest management district (LHC) (source: European Space Agency, 2024; derived from National Open Data Catalogue, 2024 and Hansen et al., 2013)

However, when focusing on the differences between the GFC and the forestry records, the surplus of the tree cover loss identified by the GFC was found to be more concentrated in specific areas. Aside from the methodological reasons already mentioned, this can also be explained by possible errors in forestry records. To better describe this hypothesis, we averaged the differences in the tree cover loss area according to the GFC and the forestry records (Fig. 9) for each forestry management district “LHC” (as shown in Fig. 12). LHCs represent the topmost spatial forestry unit for which the records are kept and strategic decisions are made by the management. The average surplus of the GFC was most pronounced in the LHC “DOBROČ” (+ 0.10), and it was also significant in the three neighbouring LHCs. To further inspect these differences, six forest units within the “DOBROČ” LHC were randomly selected, and the proclaimed tree cover losses from both data sources were visually confronted with the Sentinel 2 imagery from the fall of 2023. As shown in Fig. 13, tree cover loss was critically underestimated by the forestry records in most cases, while the GFC showed only moderate overestimation. The hypothesis questioning the accuracy of the specific logging records thus seems correct according to this example, but it would need a more systematic examination to be generally confirmed.

## Conclusions

According to our accuracy assessment, the GFC data had high producer’s and fairly good user’s accuracy of the tree cover loss detection in northern Slovakia in 2016–2022, which results in a slight overestimation. Part of the disagreement was probably caused by temporal misclassification; however, our visual interpretation confirmed limitations of the Sentinel 2 spatial resolution in depicting small, diffuse, or shrubby patches of tree cover loss.

The overestimation of tree cover loss by GFC largely occurred in the marginal pixels of its patches, as the marginality of pixels notably explained false positives of tree cover loss. This concurs with the results of other studies explaining the low accuracy of GFC by small size of tree cover loss patches. Our marginality analysis provides a broader perspective: the detection problem arises from a high ratio of pixels located at the edges of the tree cover loss patches.

Forestry logging records and GFC data show agreement on the total area of tree cover loss in 2019–2023, as well as on the general location and spatial structure of loss patches, despite differences in methodology and form of spatial representation. These datasets are therefore comparable, and both are relevant for monitoring tree cover loss in Slovakia. Nevertheless, considerable discrepancies remain in some forest management districts (LHCs), warranting caution.

In general, we regard GFC data as a valuable source for monitoring tree cover loss in Slovakia, provided its limitations and uncertainties, resulting mainly from its spatial resolution, are recognised. The release of a tree cover loss probability layer would help address this uncertainty and thus broaden the potential applications of GFC data.

## Data Availability

The source datasets are available at locations cited in references. Results of our analyses are available from the corresponding author on reasonable request.
